# Analysis of genetic diversity and structure in a worldwide walnut (*Juglans regia* L.) germplasm using SSR markers

**DOI:** 10.1371/journal.pone.0208021

**Published:** 2018-11-27

**Authors:** Anthony Bernard, Teresa Barreneche, Fabrice Lheureux, Elisabeth Dirlewanger

**Affiliations:** 1 UMR 1332 BFP, INRA, Université de Bordeaux, Villenave d’Ornon, France; 2 Ctifl, centre opérationnel de Lanxade, Prigonrieux, France; Chinese Academy of Sciences, CHINA

## Abstract

Persian or English walnut (*Juglans regia* L.), the walnut species cultivated for nut production, is one of the oldest food sources known and is grown worldwide in temperate areas. France is the 7^th^ leading producer as of 2016 with 39 kt. Deciphering walnut genetic diversity and structure is important for efficient management and use of genetic resources. In this work, 253 worldwide accessions from the INRA walnut germplasm collection, containing English walnut and several related species, were genotyped using 13 SSR (Single Sequence Repeat) markers selected from the literature to assess diversity and structure. Genetic diversity parameters showed a deficiency of heterozygotes and, for several SSRs, allele-specificities among the accessions tested. Principal Coordinate Analysis (PCoA) showed the 253 accessions clustered in largely in agreement with the existing botanical classification of the genus. Among the 217 *J*. *regia* accessions, two main clusters, accessions from Eastern Europe and Asia, and accessions from Western Europe and America, were identified using STRUCTURE software. This was confirmed by Principal Coordinate Analysis and supported by Neighbor-Joining tree construction using DARwin software. Moreover, a substructure was found within the two clusters, mainly according to geographical origin. A core collection containing 50 accessions was selected using the maximum length sub-tree method and prior knowledge about their phenotype. The present study constitutes a preliminary population genetics overview of INRA walnut genetic resources collection using SSR markers. The resulting estimations of genetic diversity and structure are useful for germplasm management and for future walnut breeding programs.

## Introduction

Persian walnut (*Juglans regia* L.) is a monoecious and dichogamous tree species [1] whose center of domestication is thought to be located in Central Asia [2]. Nowadays, Persian walnut is widely disseminated and grown in many temperate regions of Europe, North and South America, South Africa, Asia, Australia and New-Zealand [3]. The genus *Juglans* (order Fagales, family Juglandaceae) includes more than 20 diploid species, with 2n = 2x = 32 chromosomes [4]. Formerly divided into four sections–*Trachycaryon* (butternut, a single species, *J*. *cinerea*), *Cardiocaryon* (heartnuts), *Rhysocaryon* (black walnuts, notably *J*. *nigra*) and *Dioscaryon* containing only *J*. *regia* [5]–it is now suggested that *J*. *cinerea* should be included in *Cardiocaryon* section [6]. According to Food and Agriculture Organization of the United Nations and International Nut and Dried Fruit Council data (www.fao.org, www.nutfruit.org, 2014 data), world-wide in-shell walnut production exceeds 3,400 kt. The five largest producers are China, USA, Iran, Ukraine and Chile,). France is the 7^th^ largest producer with 39 kt. French walnut orchard area increased by nearly 19% between 2000 and 2010 to reach approximately 21,000 hectares in 2016, making it the most important French fruit crop other than apple. France exports 80% of its production in-shell, mainly to Europe where it is marketed in particular, thanks to the quality of its product. There are Protected Designations of Origin for the two main production areas: ‘Noix du Périgord’ and ‘Noix de Grenoble’.

Knowledge of the genetic diversity of a species is crucial for effective management and use of its germplasm. Several types of molecular markers have been used previously for assessment of diversity and relationships in other walnut germplasm or populations. For example, isozymes were used to compare European and Asian walnut genotypes [7], and to study natural populations from Italy [8]. Restriction Fragment Length Polymorphism (RFLP) markers were used to assess the genetic diversity of the genus *Juglans* [6]. Randomly Amplified Polymorphic DNA (RAPD) markers were used by the breeding program of the University of Davis, CA to analyze parents and releases [9]. RAPDs were also used to characterized Iranian genotypes [10]. Inter-Simple Sequence Repeat (ISSR) were used [11] to show the genetic diversity of Greek natural populations was greater than a comparison set of international cultivars [12]. Amplified Fragment Length Polymorphism (AFLP) analysis of walnut genotypes from Turkey showed genotypes with low chill requirement had a narrow genetic base [13]. AFLPs also were used to assess the genetic diversity of natural populations from Kurdistan [14]. Use of Simple Sequence Repeats (SSRs) has grown considerably since 2002 and more than 20 publications were recently reviewed [15].

One of the uses of genetic resources is the choice of foundation material for an improvement program. In France, the Institut National de la Recherche Agronomique (INRA–French National Institute for Agricultural Research) led a walnut breeding program from 1977 to 2005. As a first step, many crosses were performed between French cultivars (such as ‘Franquette’, with a late bud break and a good fruit quality) and California cultivars (such as ‘Pedro’ and ‘Chandler’ for their lateral bearing habit). This resulted in the release of productive cultivars with good organoleptic qualities such as: ‘Fernette’,‘Fernor’, ‘Ferjean’, ‘Feradam’, ‘Ferbel’, ‘Ferouette’ and ‘Fertignac’. Following extensive walnut germplasm collecting in the Mediterranean area, Iran, Japan, and Central Asia, additional parents containing a wider genetic base were identified and used for breeding. Although several resulting hybrids appear promising, the current context of climate change and global competition make it crucial to initiate a new improvement program, utilizing the new technologies now available.

Germplasm constitutes an essential reservoir of allelic diversity for traits currently being exploited or not yet used in breeding programs, but its preservation and management is costly [16]. Hence, creation of a core collection representing the highest genetic diversity, while decreasing the number of accessions, is an effective way to reduce costs. Consequently, INRA walnut germplasm collection, which includes 253 accessions of worldwide origin maintained at the *Prunus* and *Juglans* Genetic Resources Center, was evaluated for its genetic diversity. The objectives of this study were; 1) to assess the genetic diversity and structure of INRA walnut collection and; 2) to define a core collection suitable for future walnut breeding programs and that could be more easily managed.

## Materials and methods

### Plant material

**Fig 1:**
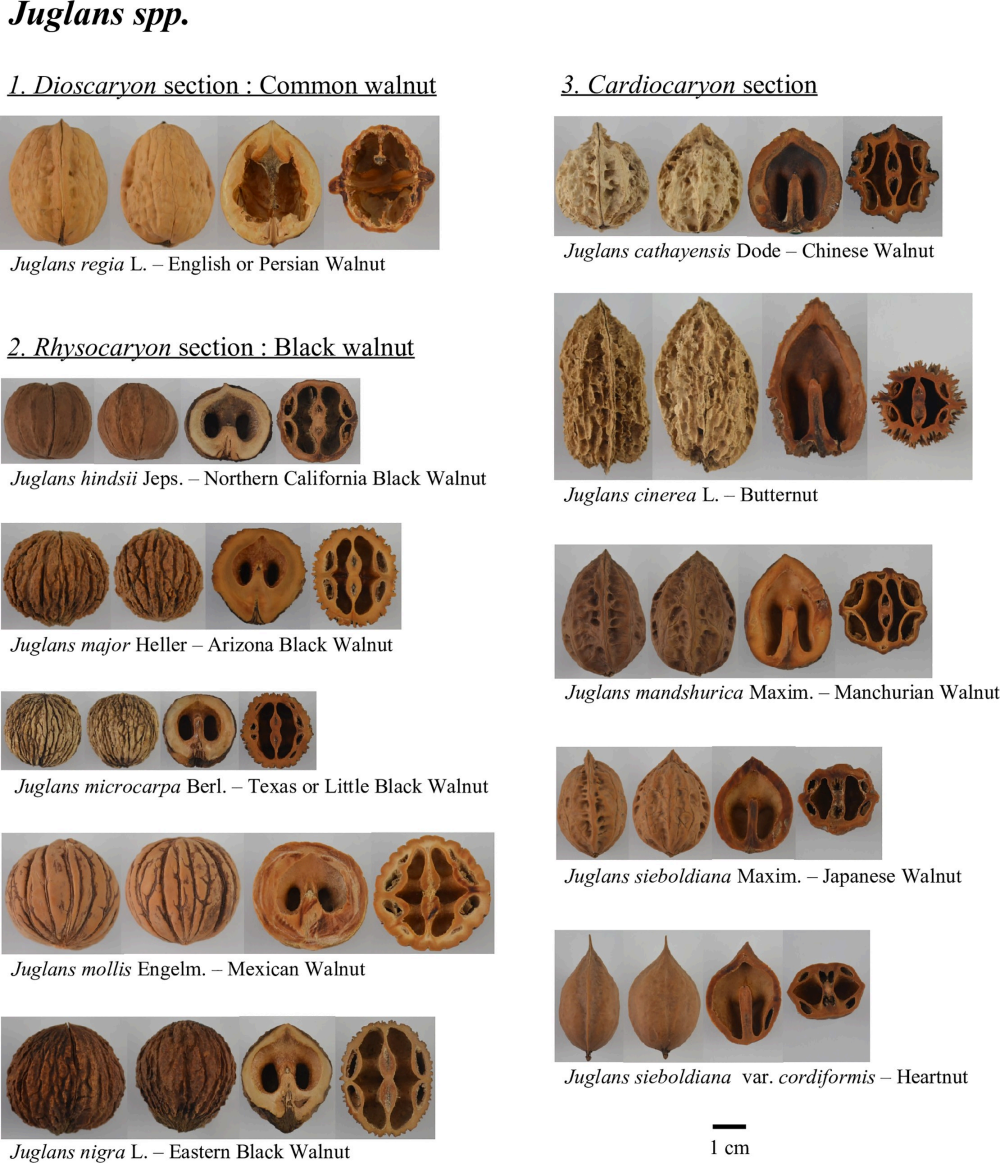
Nuts of 11 *Juglans* species available in INRA walnut germplasm collection. These *Juglans* accessions come from 23 countries covering three continents: Europe (France, Spain, Greece, Ukraine, etc.), America (Canada, USA and Chile) and Asia (China, Japan, Iran, etc.). All accessions are 20–30 year-old grafted trees on various rootstocks located in the Fruit Tree Experimental Unit (UEA) of INRA in Toulenne (latitude 44°34’37.442”N–longitude 0°16’51.48”W), near Bordeaux (France). The list of accessions studied is shown in S1 Table.

The INRA walnut germplasm collection is a result of important prospecting work performed between 1988 and 2000 in many countries around the world. 253 accessions from this collection were studied. Among them are 217 accessions of *J*. *regia*, including 194 cultivars and 23 intraspecific hybrids. An additional 36 accessions include 14 related species: *J*. *ailantifolia* Carr. (syn: *J*. *sieboldiana* Maxim.), *J*. *californica* S. Wats., *J*. *cathayensis* Dode, *J*. *cinerea* L., *J*. *hindsii* Jeps. *J*. *major* Heller, *J*. *mandshurica* Maxim., *J*. *microcarpa* Berl., *J*. *mollis* Engelm., *J*. *nigra* L., *J*. *pitteursii* C. Morren (isotype of *J*. *nigra*), *J*. *rupestris* Engelm. ex Torr. (isotype of *J*. *major*), *J*. *sieboldiana* Maxim., *J*. *sieboldiana* var. *cordiformis*. These related species accessions were added to the analysis to give a more global idea of the genetic diversity of the collection. Pictures of nuts of 11 *Juglans* species, part of the INRA walnut germplasm collection, are given in Fig 1.

### DNA extraction and SSR genotyping

The *Prunus* and *Juglans* Genetic Resources Center gave us the permission to collect the plant material. Leaves were collected and sent to the BioGEVES laboratory in France for DNA extraction using a Macherey-Nagel NucleoSpin 96 Plant II Core kit. 2 μL of DNA extraction solution was used to measure DNA concentration by spectrophotometry (SPECTROstar Omega) and extract concentrations were standardized to 2.5 ng/μL.

All accessions were genotyped using 15 SSR markers chosen from the literature [17, 18, 19], after testing initially using a small sample of accessions. The markers are listed in Table 1 with primer sequences, repeat motif, source species and references. The forward primers were tailed by addition of a 35S 19 bp oligo sequence labeled to the 5′ end, using forward primers labeled with 6-FAM, NED, PET or VIC. The PCR reactions were carried out in 10 μL volumes containing 4 μL of diluted DNA (2.5 ng/μL) and 6 μL of PCR mix. The PCR mix consisted of 3.80 μL of ultra-pure water, 1 μL of 10X buffer, 0.2 μL of dNTP (10 mM), 0.60 μL of MgCl_2_ (25 mM), 0.10 μL of Ampli Taq Gold polymerase, 0.10 μL of reverse primers (R), 1 μM of forward primers (F) and 10 μM of 35 S oligo sequence. PCR steps were: 10 min– 94°C, (30 sec– 94°C, 1 min– 58°C, 40 sec– 72°C) for 30 cycles (or 35 depending on the primers) and 10 min– 72°C. Amplification products were diluted and 5.8 μL of formamide, 0.2 μL of ladder and 4 μL of amplicon were added in each well. The SSR genotyping of all individuals was performed on an ABI 3730 DNA Analyzer (Applied Biosystems), and allele sizing was identified using the GeneMapper™ 3.7 software (Applied Biosystems), using the LIZ 500 (Applied Biosystems) as an internal size standard. To confirm SSR allele size for samples with allele size differing by only one base, a pooling of the amplicons of the samples were analyzed a second time.

**Table 1:** List of the 15 SSR markers selected.

SSR marker	SSR type and source species	Primer sequence 5'-3'	Repeat motif	Reference
WGA 001	genomic SSR from *J*. *nigra*	F ATTGGAAGGGAAGGGAAATG R CGCGCACATACGTAAATCAC	(GA)_5_GCA(GA)_3_GCA(GA)_3_	Dangl et al., 2005
WGA 004	genomic SSR from *J*. *nigra*	F TGTTGCATTGACCCACTTGT R TAAGCCAACATGGTATGCCA	(GT)_5_,(GA)_15_,(GA)_11_	Woeste et al., 2002
WGA 009	genomic SSR from *J*. *nigra*	F CATCAAAGCAAGCAATGGG R CCATTGCTCTGTGATTGGG	(GA)_16_	Dangl et al., 2005
WGA 027	genomic SSR from *J*. *nigra*	F AACCCTACAACGCCTTGATG R TGCTCAGGCTCCACTTCC	(GA)_30_	Woeste et al., 2002
WGA 069	genomic SSR from *J*. *nigra*	F TTAGTTAGCAAACCCACCCG R AGATGCACAGACCAACCCTC	(GA)_4_ATATAA(GA)_16_	Woeste et al., 2002
WGA 072	genomic SSR from *J*. *nigra*	F AAACCACCTAAACCCTGCA R ACCCATCCATGATCTTCCAA	(CT)_14_	Woeste et al., 2002
WGA 202	genomic SSR from *J*. *nigra*	F CCCATCTACCGTTGCACTTT R GCTGGTGGTTCTATCATGGG	(GA)_11_	Dangl et al., 2005
WGA 276	genomic SSR from *J*. *nigra*	F CTCACTTTCTCGGCTCTTCC R GGTCTTATGTGGGCAGTCGT	(GA)_14_	Dangl et al., 2005
WGA 349	genomic SSR from *J*. *nigra*	F GTGGCGAAAGTTTATTTTTTGC R ACAAATGCACAGCAGCAAAC	(CT)_14_	Dangl et al., 2005
WGA 376	genomic SSR from *J*. *nigra*	F GCCCTCAAAGTGATGAACGT R TCATCCATATTTACCCCTTTCG	(AG)_2_AA(AG)_6_	Dangl et al., 2005
JR 0160*	EST-SSR from *J*. *regia*	F TCTCGGATTTGGGCTGTGAC R TCCGGGACCCTCGTCTAATT	(TC)_10_	Dang et al., 2016
JR 1739*	EST-SSR from *J*. *regia*	F GGATGTGGAGACGGCAAAGA R CGTCCACCCAAACCAAGAGA	(GAGCCG)_8_	Dang et al., 2016
JR 1817	EST-SSR from *J*. *regia*	F CCTCAGAGCCAACCATCCTT R AGAACAGAACCAGCGTCACA	(AC)_11_	Dang et al., 2016
JR 6160	EST-SSR from *J*. *regia*	F ACTTCAGGTTCCCAACGCAA R TAGAGGGAAGGTCTCCGGTG	(GA)_10_	Dang et al., 2016
JR 6439	EST-SSR from *J*. *regia*	F TCGATGCGATCATCTCCGTG R CGGCACCAAAACAGAACTCG	(TGCG)_5_	Dang et al., 2016

F: Forward, R: Reverse

*: not retained for the analysis

### Assessment of genetic diversity and structure

Measures of genetic diversity, including the total number of observed alleles (A), the observed heterozygosity (H_o_), the expected heterozygosity (H_e_) and the inbreeding coefficient (F_IS_) were estimated for each locus with the “adegenet 2.1.0” R package [20], for the 217 *J*. *regia* accessions only. The number of effective alleles (A_e_) was estimated using GenAlEx 6.5 software [21, 22] and the polymorphism information content (PIC) was calculated using Cervus 3.0 software [23]

To determine the relationships between *Juglans* species, Principal Coordinate Analysis (PCoA) was used. PCoA is a distance-based model using jointly a dissimilarity matrix calculated with a simple-matching index and a factorial analysis. PCoA was performed using DARwin 6.0.14 software (Dissimilarity Analysis and Representation for Windows) [24] supplemented by “scatterplot3d” R package for 3D visualization, to determine the relationships between the different *Juglans* accessions.

PCoA was used to investigate the patterns of structure among the 217 *J*. *regia* accessions and Bayesian model-based analyses was implemented using the software package STRUCTURE 2.3.4 [25]. The following steps were performed to identify the best number of clusters (K): twenty runs of STRUCTURE were done by setting K from 1 to 10. Each run consisted of a length of burn-in period of 5,000 followed by 50,000 Markov Chain Monte Carlo (MCMC) replicates, assuming an admixture model and correlated allele frequencies. When K was estimated, ten runs of STRUCTURE were done by setting the K from 1 to 5. Each run consisted of a length of burn-in period of 100,000 followed by 750,000 MCMC replicates, assuming an admixture model and correlated allele frequencies. For the choice of the most likely K, the plateau criterion described by Pritchard et al. [25] and the ΔK method described by Evanno et al. [26], implemented in STRUCTURE harvester [27], were used. Then, the run having the highest likelihood estimate to assign cluster proportions to individuals was used. Accessions with an estimated membership below 0.8 were assigned to the “admixed group”. Then, STRUCTURE was run in a second step on fragmented datasets to discover if lower levels of structure exist and K was tested from 1 to 10 for each cluster (ten runs, 5,000 burn-in period, 50,000 MCMC replicates) and when K was estimated, ten runs were done by setting the K from 1 to 5 (100,000 burn-in period, 750,000 MCMC replicates). The ΔK method described by Evanno et al. [26], implemented in STRUCTURE harvester [27], was used.

In addition, the pairwise fixation indexes (F_ST_) between clusters were estimated with the “diveRsity 1.9.90” R package [28]. The genetic relationships between *J*. *regia* accessions was also assessed by the Neighbor-Joining method [29] using DARwin 6.0.14 software. Dissimilarities were calculated with 10,000 bootstraps, transformed into Euclidean distances using power transformation, and the UnWeighted Neighbor-Joining method was used to build a tree for *J*. *regia* accessions.

### Core collection establishment

Core collections are subsamples of germplasm collections created in order to decrease the number of accessions while still representing the maximum genetic diversity of the larger collection. The function ‘maximum length sub tree’ of DARwin 6.0.14 software [24] was used to create a *J*. *regia* core collection based on dissimilarities calculated as described before. This is a stepwise procedure that prunes successively redundant accessions, allowing the choice of sample size that retains the largest genetic diversity. Putative clusters of synonymous accessions were found using the function ‘removed edge value’ provided by the Neighbor-Joining tree with a threshold value of 0.0005. A core collection must also account for other knowledge available such as the adaptation of an accession to a particular environment or its interest in terms of other phenotypes. In the ‘maximum length sub tree’ function, the ‘excluded’ and ‘forced’ options allow excluding or forcing inclusion of an accession manually. This option was used to keep several reference accessions in the final core collection.

## Results

### SSR genotyping and genetic diversity analysis

According to the amplification control, the strength of recorded signal and the number and quality of true peaks, 13 SSR markers among the 15 were selected for analysis of the 253 accessions. In addition, because each marker was carefully developed and tested, we assumed that the missing data are in fact null alleles. For several SSRs, PCR amplification was observed only for a portion of the *Juglans* species tested (S2 Table). The WGA 001 and WGA 276 SSRs amplify in *J*. *regia* and species of section *Rhysocaryon* but not in section *Cardiocaryon* (S2 Table). The JR 1817 SSR does not amplify in *Cardiocaryon* or in *J*. *californica* but does amplify in the other *Rhysocaryon* and in *J*. *regia*. The JR 6160 SSR also does not amplify in *J*. *californica* (S2 Table). Furthermore, for some SSRs, private alleles are observed in some species. For example, for WGA 001 the ‘184 bp’ allele is found only in *J*. *mollis* and the ‘197 bp’ and ‘205 bp’ alleles are present exclusively in *J*. *nigra*. For WGA 009, the ‘247 bp’ allele is only detected in *J*. *regia*, in *Cardiocaryon* species, and in *J*. *nigra* and *J*. *pitteursii* of section *Rhysocaryon* and for WGA 069, the ‘180 bp’ allele is found only in *J*. *regia* and section *Cardiocaryon*. Finally, for WGA 376 the ‘236 bp’ allele is present only in *J*. *regia* and section *Rhysocaryon* whereas the ‘244 bp’ allele is present only in section *Cardiocaryon*.

**Fig 2:**
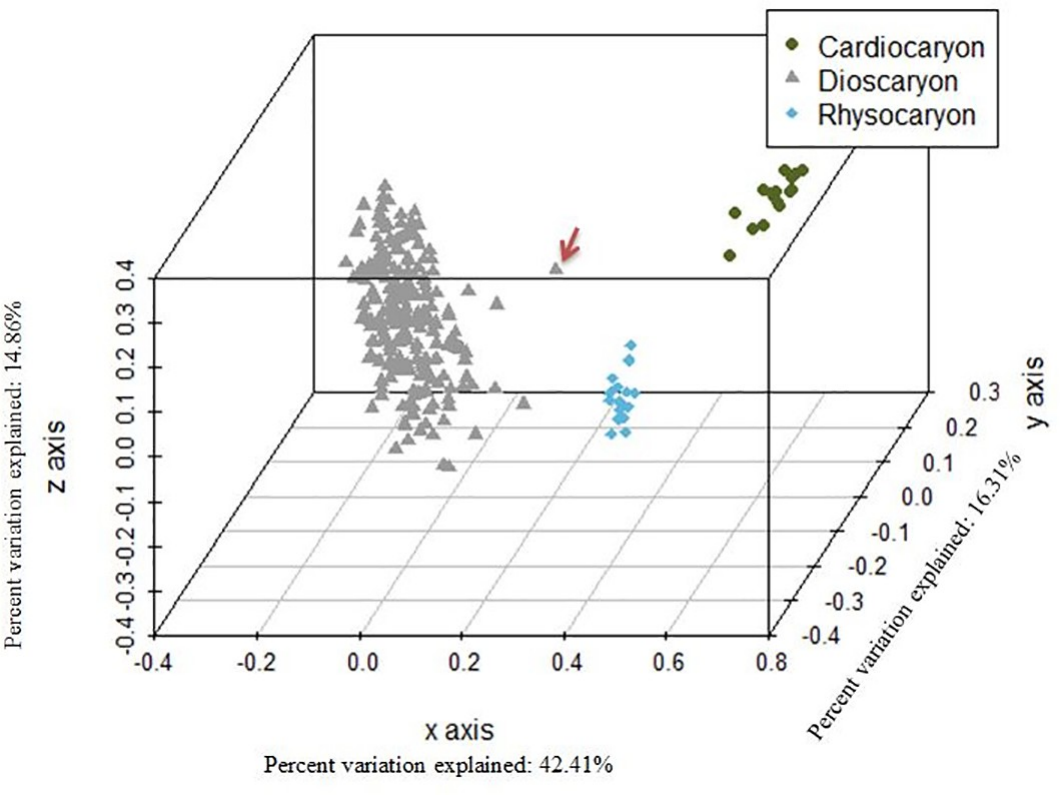
Principal Coordinate Analysis (PCoA) 3D plot of the 253 *Juglans* accessions using 13 SSRs. Blue cluster shows the 18 accessions belonging to the *Rhysocaryon* section while the green cluster shows the 18 accessions belonging to the *Cardiocaryon* section, both part of the *Juglans* genus. In grey, *J*. *regia* accessions (*Dioscaryon* section). The appearing distant *J*. *regia* accession marked with a red arrow is the accession ‘PI 15 95 68’ (ID 207).

PCoA is a method that explores dissimilarities of data through a dissimilarity matrix and assigns each accession a location in a low-dimensional space. The PCoA results reveal three well-separated clusters among INRA *Juglans* germplasm collection (Fig 2). The largest cluster contains the 217 *J*. *regia* accessions. A second cluster consists of the *Cardiocaryon* section and a third contains the *Rhysocaryon* species. The *Cardiocaryon* cluster includes 18 accessions of *J*. *mandshurica*, *J*. *cathayensis*, *J*. *sieboldiana*, *J*. *sieboldiana* var. *cordiformis*, and *J*. *cinerea*. The *Rhysocaryon* cluster includes 18 accessions of *J*. *mollis*, *J*. *nigra*, *J*. *microcarpa*, *J*. *californica*, *J*. *hindsii* and *J*. *major*.

Among the 217 *J*. *regia* accessions, the number of alleles per locus (A) ranges from 2 to 17 with an average of 8.92 and the number of effective alleles ranges from 1.18 to 4.61 with an average of 2.84 (Table 2). The observed heterozygosity (H_o_) ranges from 0.10 to 0.68 with a mean value of 0.47. The expected heterozygosity H_e_ is higher than H_o_ and ranges from 0.15 to 0.78 with a mean value of 0.56 (p-value of *t*-test: 8.24E^-04^). The average polymorphism information content (PIC) is close to that of H_e_. JR 1817 and JR 6439, both SSRs derived from EST, have the lowest values of H_o_, H_e_, A_e_ and PIC. Considering all the accessions including related species, the number of alleles per locus (A) is larger at each locus, ranging from 7 to 32 with an average of 17.31.

**Table 2:** Genetic diversity estimations of the 217 *J*. *regia* accessions.

SSR markers	A	A_e_	H_o_	H_e_	PIC	F_IS_
WGA 001	8	3.50	0.65	0.71	0.67	0.08
WGA 004	7	1.99	0.46	0.50	0.42	0.08
WGA 009	7	2.93	0.59	0.66	0.62	0.11
WGA 027	2	1.87	0.43	0.46	0.36	0.07
WGA 069	9	4.25	0.57	0.76	0.73	0.25
WGA 072	6	1.73	0.32	0.42	0.37	0.24
WGA 202	17	4.61	0.68	0.78	0.75	0.13
WGA 276	16	4.15	0.63	0.76	0.74	0.17
WGA 349	11	4.59	0.49	0.78	0.75	0.37
WGA 376	13	2.95	0.61	0.66	0.62	0.08
JR 1817	5	1.18	0.10	0.15	0.15	0.33
JR 6160	10	1.96	0.43	0.49	0.42	0.12
JR 6439	5	1.21	0.14	0.17	0.17	0.18
*Min*	2	1.18	0.10	0.15	0.15	0.07
*Mean*	8.92	2.84	0.47	0.56	0.52	0.17
*Max*	17	4.61	0.68	0.78	0.75	0.37

A: number of different alleles, A_e:_ number of effective alleles, H_o_: observed heterozygosity, H_e_: expected heterozygosity, PIC: polymorphism information content, F_IS_: inbreeding coefficient

### Structure analysis

**Fig 3:**
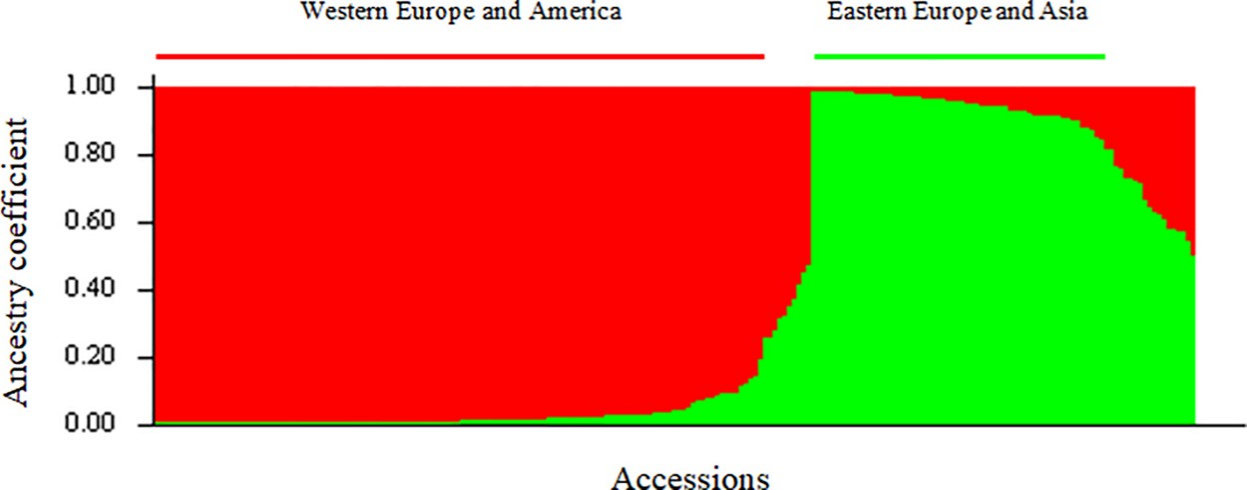
Structure of the *J*. *regia* collection using STRUCTURE software. Bar plot of individual ancestry proportions for the genetic clusters inferred using K = 2 and the dataset of 217 individuals and 13 SSR loci. Individual ancestry proportions (Q values) are sorted within each cluster. ‘Eastern Europe and Asia’, and ‘Western Europe and America’ clusters, are shown in green and red, respectively.

Analysis of structure infers accession ancestry from genotypic information. The most likely number of clusters was evaluated considering the plateau criterion and using the ΔK method. The highest value was for K = 2 (S3 Table; S1 Fig), indicating two clusters: the first containing 63 accessions (referred to as the ‘Eastern Europe and Asia’ cluster). The second comprises 127 accessions (the ‘Western Europe and America’ cluster) (Fig 3; S4 Table). An additional 27 accessions clearly showed mixed ancestry (membership values lower than 80% in either of these two clusters). In this admixed cluster, 17/27 accessions are INRA hybrids and USA cultivars resulting from intraspecific crosses (‘Feradam’, ‘Fernette’, ‘Serr’, ‘Chico’, ‘Amigo’, ‘Gillet’, ‘Forde’ and ‘Tulare’). The two accessions with purple leaves are also in this admixed group, as well as other accessions, including ‘Lara’.

The ‘Eastern Europe and Asia’ cluster contains accessions from Bulgaria (‘Sheinovo’ and ‘Izvor 10’), Romania (‘Sibisel 39’, ‘Sibisel 44’, ‘VL4B’ and ‘Germisara’), Greece (‘S 28 A Achille’, ‘S 4 B Thétis’, ‘S 34 B Pyrrus’ and ‘EAA 6’), Hungary (‘Milotai n°10’), Ukraine (‘UK-series’), India (‘Sopore’), Iran (‘IR-series’ and ‘Z 53’), China (‘Jin Long 1’ and ‘Lu Guang’), Japan (‘Shinrei’), and surprisingly, France (some INRA hybrids), Switzerland (‘RA 1223’) and USA (‘Sexton’, ‘Chase C7’, ‘Wepster W2’, ‘Adams 10’ and the ‘PI series’).

The ‘Western Europe and America’ cluster contains accessions from France (old cultivars such as ‘Lub’, ‘Franquette’, ‘Saint Jean’, ‘Marbot’, ‘Bijou’, modern cultivars obtained from intraspecific crosses such as ‘Fernor’, ‘Ferjean’, ‘Ferbel’, ‘Ferouette’, ‘Fertignac’, and INRA hybrids), Germany (‘Allemagne 139’ and ‘Geisenheim 286’), England (‘Northdown Clawnut 252’), Spain (‘MB- and MBT-series’, ‘Del Carril’ and ‘Gran Jefe’), Greece (‘S 1 A Diane’ and ‘S 1 B Ariane’), Portugal (‘Rego’), Switzerland (such as a laciniate walnut), Hungary (‘M 10–37’), Chile (‘AS 1’) and USA (old cultivar ‘Payne’ and modern cultivars obtained from intraspecific crosses such as ‘Howe’, ‘Pedro’, ‘Vina’, ‘Chandler’, etc.).

Moreover, genetic diversity parameters show a lower level of diversity among the ‘Western Europe and America’ cluster (S7 Table) and pairwise F_ST_ calculation between the two clusters is higher than that between each of the two clusters and the admixed group (S8 Table). Among the 217 *J*. *regia* accessions, a higher number of private alleles was observed in the ‘Eastern Europe and Asia’ cluster compared to the number in the ‘Western Europe and America’ cluster (61 alleles vs 5 alleles) (S9 Table).

**Fig 4:**
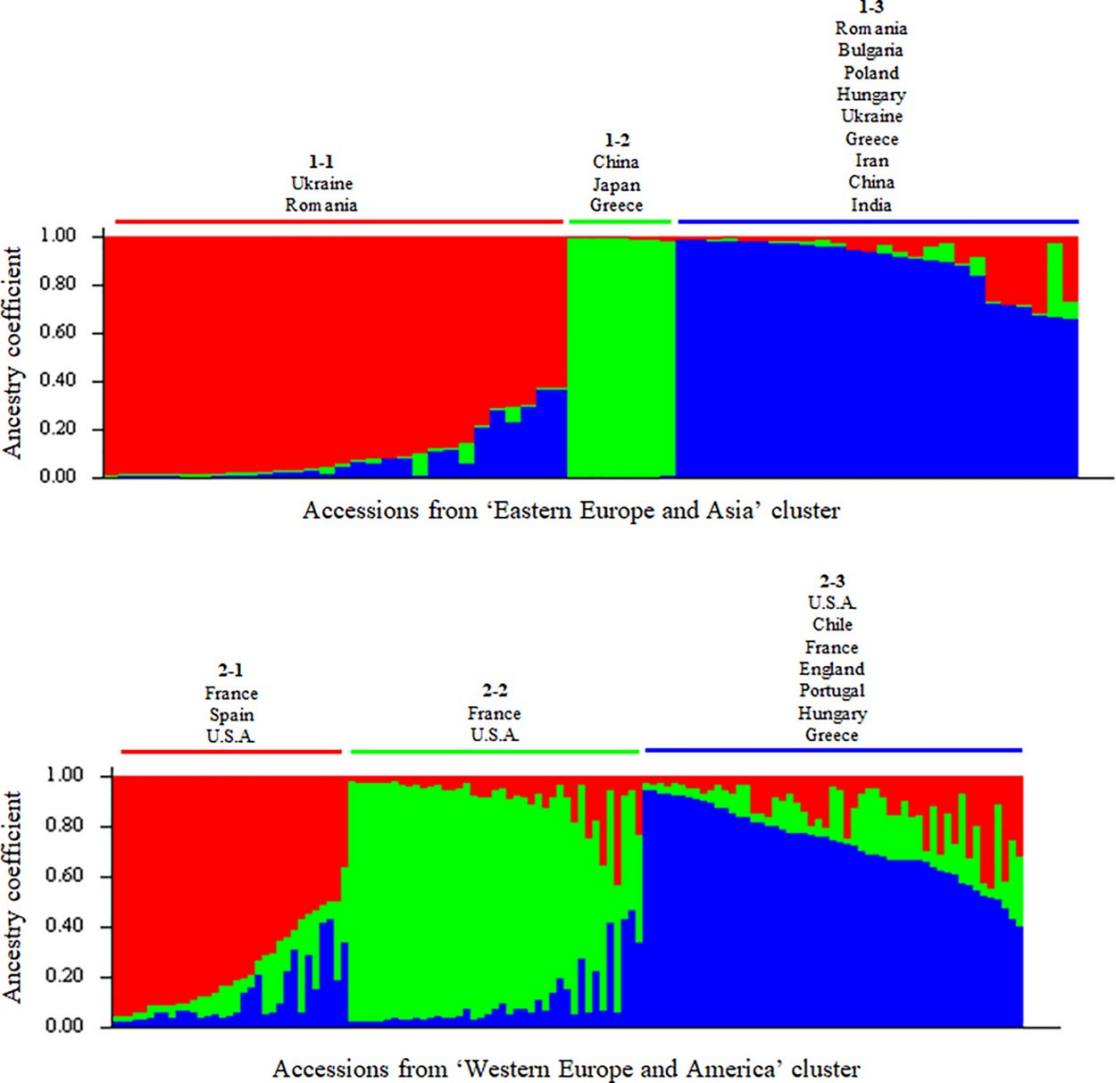
Substructure of the ‘Eastern Europe and Asia’ and ‘Western Europe and America’ clusters using STRUCTURE software. Bar plot of individual ancestry proportions for the genetic clusters inferred, using K = 3 and the dataset of 63 individuals and 13 SSR loci, for the ‘Eastern Europe and Asia’ cluster at the top. Bar plot of individual ancestry proportions for the genetic clusters inferred, using K = 3 and the dataset of 127 individuals and 13 SSR loci, for the ‘Western Europe and America’ cluster at the bottom. Individual ancestry proportions (Q values) are sorted within each cluster. Substructure within each of the two large groups follows a geographical origin, based on the supposed origin of the accessions.

As the ΔK method often detects the uppermost level of structure of a collection, each cluster was analyzed independently to explore if a lower structure could be found within each group. The two separated datasets comprised 63 accessions coming from ‘Eastern Europe and Asia’ cluster, and 127 accessions coming from ‘Western Europe and America’ cluster. The 27 accessions considered as admixed were excluded from further analyses. Within each cluster, STRUCTURE identified three subclusters (Fig 4). Among the subclusters of ‘Eastern Europe and Asia’, 1–1 contains 29/31 accessions of the ‘UK-series’ from Ukraine and one accession from Romania (‘VL4B’). Subcluster 1–2 contains one Greek accession (‘EAA 6’), one Chinese accession (‘Jin Long 1’), the Japanese accession (‘Shinrei’), and unexpectedly a French INRA’s hybrid and three accessions from USA (‘Chase C7’, ‘Wepster W2’ and ‘Adams 10’). Subcluster 1–3 contains accessions from Asia including Chinese accession ‘Lu Guang’ and Indian accession ‘Sopore’, accession ‘RA 1223’, the two other accessions (2/31) from Ukraine, and several accessions from eastern European countries such as Greece, Romania, Bulgaria, Poland and Hungary. (S5 Table; S2 Fig). Subcluster 2–1 of the ‘Western Europe and America’ cluster includes old French cultivars such as ‘St Jean’, ‘Corne’, ‘Grosvert’, ‘Bijou’ and ‘Verdelet’, accession ‘Lieb Mayette’, all the Spanish accessions, some accessions from USA and several others including the laciniate walnut. Subcluster 2–2 includes other old French cultivars (‘Meylannaise’, ‘Franquette’ and ‘Mayette’) and accessions from USA (‘Pedro’, ‘Midland’, ‘Vina’, ‘Cisco’, ‘Chandler’ and ‘Carmelo’) and subcluster 2–3 includes other old French cultivars (‘Lozeronne’, ‘Parisienne’ and ‘Marbot’), accessions from USA (‘Tehama’ and ‘Waterloo’), Chilean accession ‘AS 1’, and accessions from England, Portugal, Hungary and Greece (S6 Table; S3 Fig).

**Fig 5:**
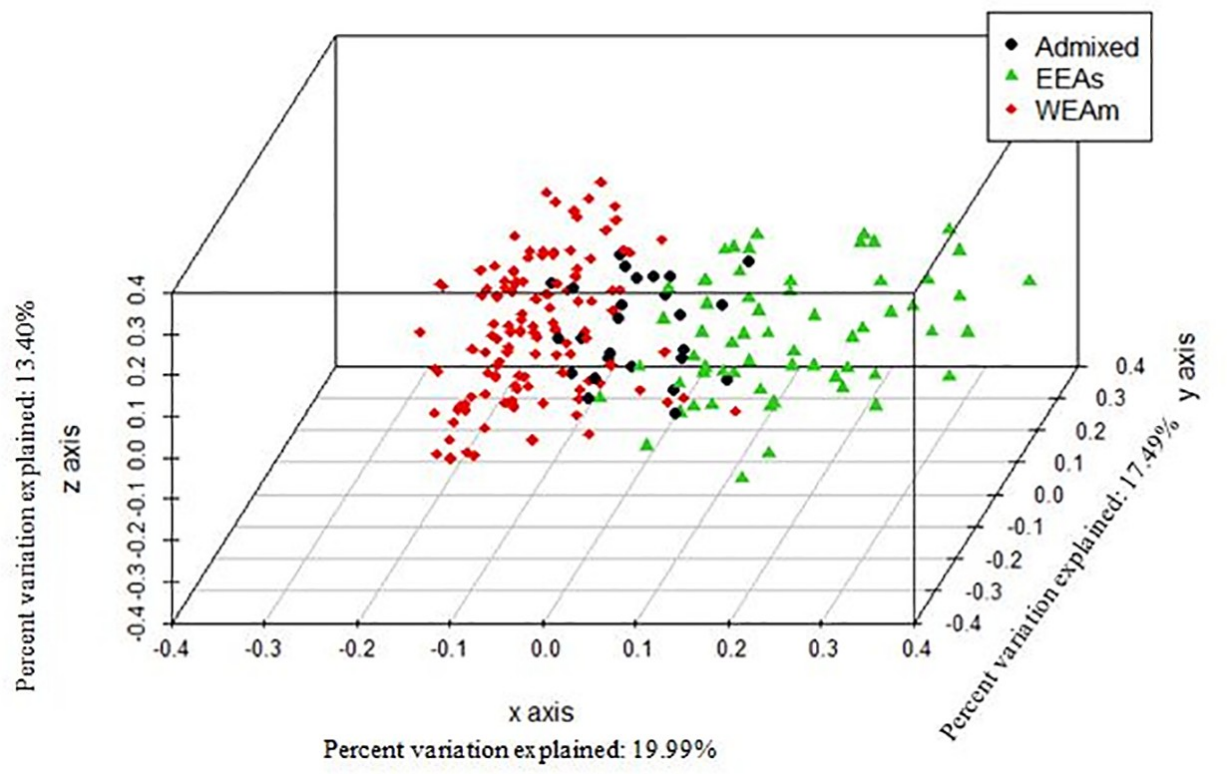
Principal Coordinate Analysis (PCoA) 3D plot of the 217 *J*. *regia* accessions from the INRA walnut germplasm collection using 13 SSRs. Colors indicate STRUCTURE clusters K = 2: red for ‘Western Europe and America’ (WEAm) cluster, green for ‘Eastern Europe and Asia’ (EEAs) cluster, and black for admixed accessions.

PCoA performed on the set of 217 *J*. *regia* accessions (Fig 5) showed very similar clustering to those obtained with STRUCTURE. Each cluster again separated distinctly although the ‘Eastern Europe and Asia’ accessions were more spread, suggesting a greater genetic diversity.

**Fig 6:**
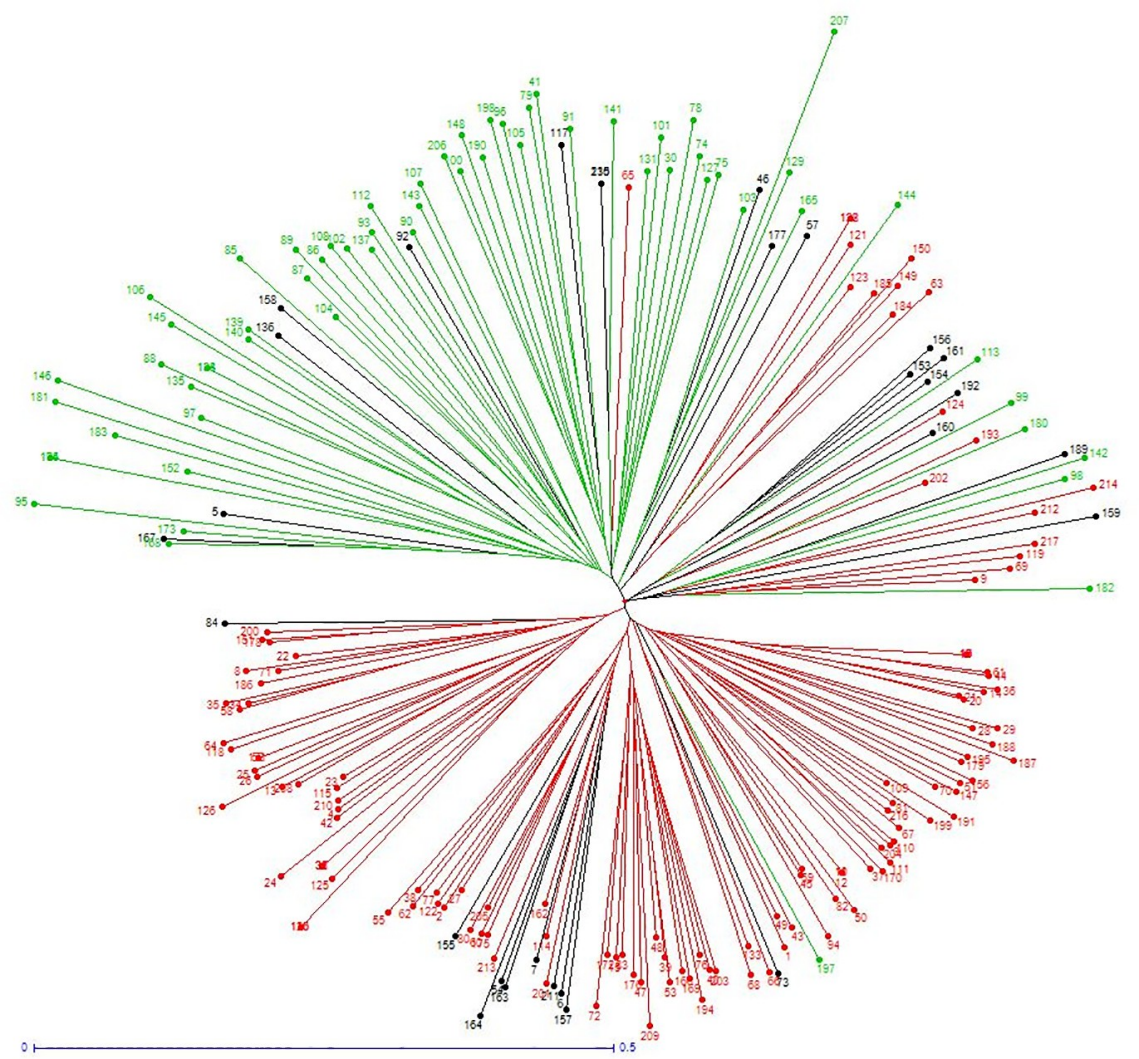
Neighbor-Joining tree based on dissimilarity matrix calculated from the dataset of 13 SSRs across the 217 *J*. *regia* accessions from the INRA’s walnut germplasm collection. Colors reflect STRUCTURE clusters K = 2: red for ‘Western Europe and America’ cluster, green for ‘Eastern Europe and Asia’ cluster, and black for admixed accessions.

Results obtained with STRUCTURE for *J*. *regia* accessions generally also aligned with assessment using the Neighbor-Joining method (Fig 6). The main branching groups of the tree agree with STRUCTURE results with K = 2. Several accessions exhibit longer branch length, indicating a greater genetic diversity. These include accessions ‘PI 15 95 68’ (ID 207), ‘Sopore’ (ID 181), ‘Jin Long 1’ (ID 95) and ‘UK 239–23’ (ID 146), which belongs to ‘Eastern Europe and Asia’ cluster. This finding is in agreement with the potential greater level of genetic diversity of the accessions of ‘Eastern Europe and Asia’ cluster found using PCoA.

### Core collection establishment

**Fig 7:**
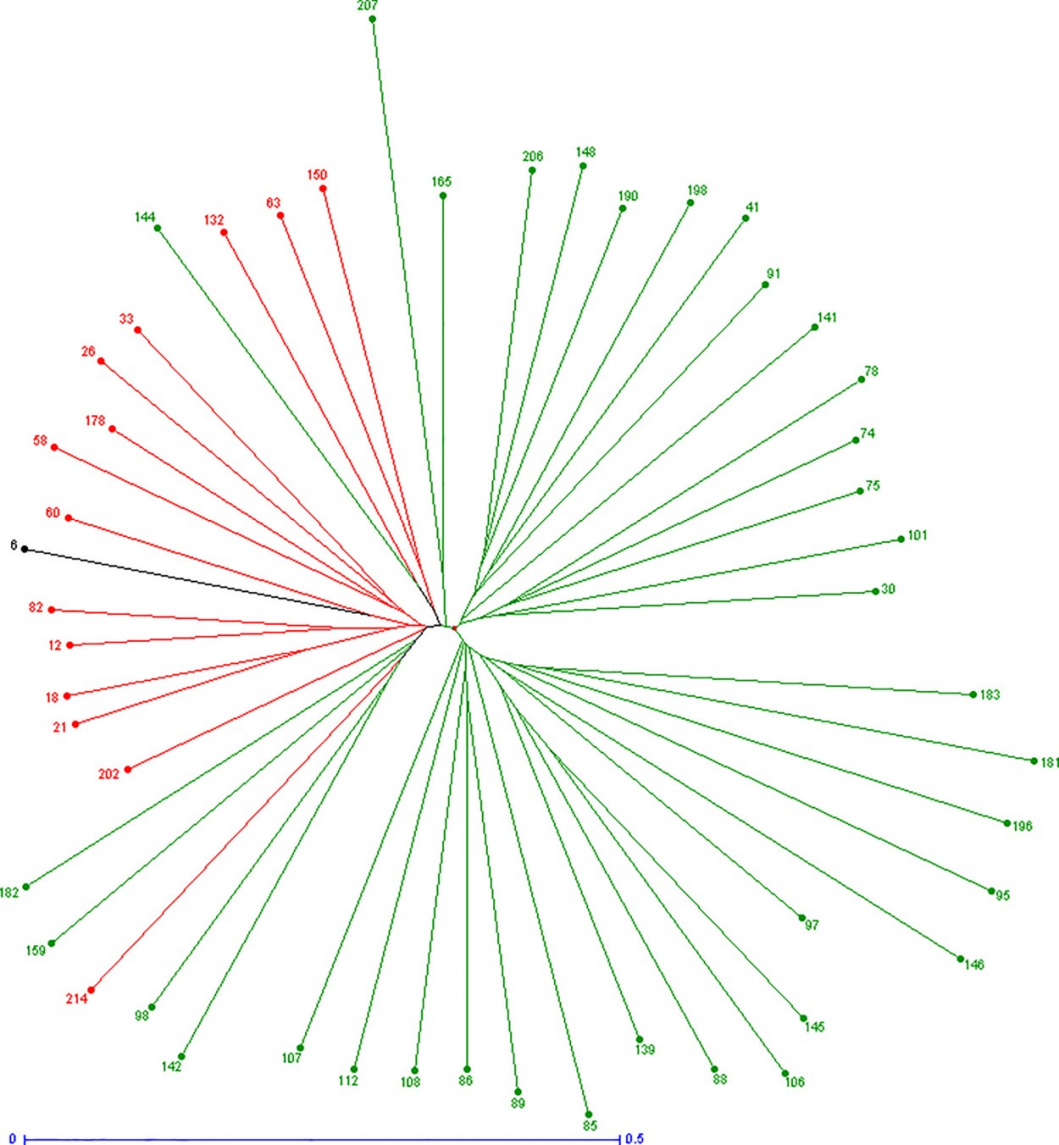
Neighbor-Joining tree of the accessions chosen to construct the INRA walnut germplasm core collection. Colors correspond to STRUCTURE clusters K = 2: red for the ‘Western Europe and America’ cluster, green for the ‘Eastern Europe and Asia’ cluster, and black for admixed accessions.

The Neighbor-Joining tree based on the dissimilarity matrix between the 217 *J*. *regia* accessions (Fig 6) allowed construction of a core collection. The function ‘maximum length sub tree’ of DARwin 6.0.14 software was used iteratively to eliminate the putatively synonymous and the most redundant accessions. Additionally, some accessions were fixed based on knowledge of the material, using the DARwin ‘forced’ option. This revision of the Neighbor-Joining tree is shown in Fig 7.

This study led to the choice of 50 accessions for a core collection (S4 Table). Among these, 11 were fixed selections and six were retained for their phenotypic traits or adaptability. This set included ‘Chandler’, the most important cultivar in California and also cultivated in France, ‘Early Ehrhardt’, the earliest to break bud, ‘Bijou’, with the biggest fruits, ‘Franquette’ and ‘Lara’, among the most planted cultivars in French orchards, and ‘Rouge de la Réole’, for its red kernels. Five additional accessions were selected because they belong to the two French Protected Designations of Origin for the two main production areas: ‘Noix du Périgord’ cultivars ‘Grandjean’, ‘Corne’ and ‘Marbot’, and ‘Noix de Grenoble’ cultivars ‘Mayette’ and ‘Parisienne’. With the exception of ‘Lara’, which is admixed, all these accessions are part of the ‘Western Europe and America’ cluster. In Fig 7 they form the red cluster from ID 33 to ID 202.

The remaining 39 accessions include only four accessions from the ‘Western Europe and America’ cluster, in particular a laciniate leafed walnut and the Greek accession ‘S 1 B Ariane’. The remaining 35 are part of the ‘Eastern Europe and Asia’ cluster, and include 17 ‘UK-series’, the three ‘PI-series’, five Iranian genotypes, Chinese cultivar ‘Jin Long 1’, the Greek ‘EAA 6’, the Indian ‘Sopore’, ‘Germisara’, ‘Sibisel 44’ and ‘Sibisel 39’ from Romania, ‘Izvor 10’ and ‘Cheinovo’ from Bulgaria, ‘Sexton’, and an ornamental tree.

## Discussion

### Genetic diversity of the INRA walnut germplasm collection

This study provides the first overview of genetic diversity in a large germplasm collection of walnut. Although WGA SSRs are derived from *J*. *nigra*, they have good transferability to *J*. *regia*. The two EST-SSRs derived from *J*. *regia*, JR 1817 and JR 6439, have the lowest H_o_ and H_e_ values. EST-SSR polymorphism reflects the genetic diversity in coding sequences. The low polymorphism observed may be due to selection against alteration in conserved coding sequences. This was also observed in grape [30], cucumber [31] and various other plant species [32].

The three clusters revealed by PCoA (Fig 2) closely follow the botanical classification of the species of genus *Juglans* as previously observed using RFLPs [6] and nuclear DNA sequences [33]. In this study, section *Rhysocaryon* seems to be closer to *Juglans regia* than is the *Cardiocaryon* section. This contrasts with other evidence *Cardiocaryon* is the section most closely related to *J*. *regia* [3]. This could be a result of the small number of accessions of these two sections included for analysis or to unsuccessful amplification of SSRs WGA 001 and WGA 276 in the *Cardiocaryon* section.

Expected heterozygosity (H_e_) of the 217 *J*. *regia* accessions was higher than observed heterozygosity (H_o_), showing a deficiency of heterozygotes that suggests the presence of pedigree inbreeding. Average H_o_ (0.47) and average H_e_ (0.56) are similar to those found in other studies on walnut natural populations or germplasm collections, with a lower H_o_ [34–44] and indicates a potential structure within the INRA walnut germplasm collection.

### Structure and genetic relationships of the INRA walnut germplasm collection

The diversity of the INRA *J*. *regia* germplasm collection, in terms of geographical origin, is a result of international cooperation and collections by Eric Germain, the former INRA walnut breeder. He travelled extensively and collected diverse plant materials from both natural populations and the collections of foreign breeding programs. Regarding the structure of the collection, the Bayesian model-based STRUCTURE method and the PCoA gave similar results, as was also observed in coconut data [45]. The INRA collection consists of two well-differentiated clusters, as is often reported in other plant germplasm collections, for example, soybean, cherry, maize, apple and coffee [46–50]. The two genetic clusters of the INRA walnut collection fit well with the geographical origin of the accessions. Results were consistent with Pollegioni et al. [51] regarding the history of *J*. *regia* in Europe. That work showed that the genetic structure of 91 walnut populations in Eurasia separated into two main clusters, Western Europe (Italy, Spain, France, Slovakia and Hungary) and Eastern Europe and Asia (Romania, Greece, Turkey, Iran and the Himalayas). Similar results were found for sweet chestnut, with a strong differentiation between trees from the Northern Iberian Peninsula, and Central and Southern Iberian Peninsula, as well as in natural populations [52], and among European cultivars [53, 54]. All walnut accessions in the ‘admixed group’ are known hybrids. It includes the French variety ‘Lara’, the result of a complex cross: ['Hartley' × 'Payne'] × ['PI 18256' × 'Conway Mayette']. The nine ‘admixed’ INRA hybrids, for example ‘Fernette’, have ‘Lara’ in their pedigree. The six other ‘admixed’ modern cultivars are the American accessions ‘Serr’ (a cross between ‘Payne’ and ‘PI 15 95 68’ from Afghanistan), ‘Chico’ and ‘Amigo’ (resulting both from a cross between ‘Sharkey’, maybe with Chinese pedigree, and ‘Marchetti’), ‘Tulare’ (with ‘Serr’ parentage), and ‘Gillet’ and ‘Forde’ (both with ‘Chico’ parentage). Therefore, it is not surprising to obtain lower values of pairwise F_ST_ between the ‘admixed’ and ‘Western Europe and America’ clusters, and between the ‘admixed’ and ‘Eastern Europe and Asia’ clusters. Interestingly, the unique Israeli accession ‘Kfar Hanania’ shares its genome with both clusters at 50%.

The genetic diversity parameters H_e_, H_o_, F_IS_ and the number of alleles are lower among the 127 ‘Western Europe and America’ accessions than among the 63 of the ‘Eastern Europe and Asia’ cluster (S7 Table). This is in agreement with the fact that walnut was domesticated in Central Asia [2], and that regions of origin are expected to contain larger genetic diversity. In addition, these results show that the accessions of the ‘Western Europe and America’ cluster, in particular those from France and USA, might in fact be more related than expected under a model of random mating. Furthermore, the pairwise fixation index (F_ST_) calculated between the two clusters shows a clear level of differentiation, with a value of 0.101 (S8 Table). A search of private alleles also shows a greater allelic diversity in the ‘Eastern Europe and Asia’ cluster even though there are half as many individuals in this cluster as in the other (S9 Table). Investigation of substructure among each of the two main clusters gives a good understanding of the plant material. Within the ‘Eastern Europe and Asia’ cluster, the subclustering also follows the geographical origins. Accessions of the ‘UK-series’ (subcluster 1–1, Fig 4), from a likely center of origin, were well separated from those of Eastern Europe, Iran and India (1–3). A small subcluster (1–2) contained both accessions from the Far East and Greek accession ‘EAA 6’. According to the plant material register of E. Germain, this accession was collected in Central Greece but it seems to be different from other Greek accessions. In the same way, the subclustering within the ‘Western Europe and America’ group follows the geographical origin of accessions. Spanish accessions (2–1) are separated from Greek accessions (2–3). French accessions, highly represented in this study, are scattered throughout the three subclusters accordingly to their regional origin. For example, the landraces ‘Corne’, ‘Grosvert’, ‘Verdelet’ and ‘Saint Jean’ belong to the 2–1 group. These are from the Dordogne or Corrèze departments in Nouvelle-Aquitaine region (western France), one of the main French walnut production regions, whereas the landraces ‘Meylannaise’, ‘Mayette’ and ‘Franquette’, which are part of 2–2 group, come from the Isère department in the Auvergne-Rhône-Alpes region (eastern France), another important region of walnut production. The 2–3 group consists of other French landraces including ‘Candelou’ from the Lot department in the Occitanie region. So, even among a relatively small number of accessions, the French landraces show a level of genetic diversity corresponding to their geographical origin.

The structure observed in INRA walnut collection provides interesting insights into source materials and the pedigrees of hybrids and cultivars developed by past breeding programs, and provides information for the better use of germplasm collections, including for the choice of parents, for the new French walnut breeding program in particular [15]. For example, the INRA hybrids H 91–88 (‘Franquette’ × ‘Payne’), H 93–63 (‘Franquette’ × ‘Pedro’), and H 99–104 (‘Franquette’ × ‘Chandler’) and their progenitors belong to the ‘Western Europe and America’ cluster, along with all old French cultivars. Conversely, USA accessions ‘Chase C7’, ‘Wepster W2’ and ‘Adams 10’ belong to the ‘Eastern Europe and Asia’ cluster, probably because they are selections of ‘Manregian-type’ walnuts. ‘Manregian’ walnuts trees originate from seed collected in northeastern China. Accessions ‘PI 2 657 12’ and ‘PI 15 95 68’, respectively from Russia and Afghanistan, also belong to the ‘Eastern Europe and Asia’ cluster. Indeed, in INRA walnut collection, we have the information of the source country which provided us the accessions. But the source country could be different from the real geographical origin of the accessions and this work gives us new information on the plant material. Furthermore, even if it is important to note that the INRA walnut collection includes a lot of French and American selections or hybrids, for some they derive from crosses using parents from various countries of origin.

Some INRA *J*. *regia* accessions show interesting or unusual traits such as weeping branches or laciniate leaves and have been used for ornamental purposes. There are also four accessions of ‘red walnuts’. These have red pellicles. Pellicle coloration in general is an important commercial trait. Lighter kernels command higher prices but at a time when consumers are looking for diversity on their plates, such as the black tomato, red walnuts could become a new trend. The genetic determinant of kernel pellicle coloration was identified recently [55, 56]. In this study, four accessions ‘Robert Livermore’ (ID 200), ‘Rouge de la Donau’ (ID 185), ‘Rouge de la Réole’ (ID 178) and ‘Rouge de Laquenexy’ (ID 179) were studied. Results found with STRUCTURE K = 2 and DARwin show a high degree of similarity between ‘Robert Livermore’ and ‘Rouge de la Réole’, explained by the fact that ‘Robert Livermore’ is derived from a controlled cross between ‘Howard’ and ‘Rouge de la Réole’, the latter having been introduced under the name ‘UC86-11’ into the California germplasm [57]. Moreover, according to the Neighbor-Joining tree, the two other accessions seem to be genetically distant, probably due to their assumed origin: ‘Rouge de la Donau’ from Austria and ‘Rouge de Laquenexy’ that originated close to Metz, near the German border. The nature of available diversity is interesting if future selection for this trait is needed.

### Core collection

Results of the Neighbor-Joining method supported those found with STRUCTURE and PCoA. The constructed tree identified 18 putative synonyms. These include the three ‘Corne’ accessions and three ‘Parisienne’ accessions. A core collection should conserve the maximum phenotypic and genotypic variation in as few accessions as possible [58], avoiding redundant entries and including all relevant geographical regions [59]. This study proposes the first core collection for INRA walnut germplasm collection based on genotypic variation and additional knowledge of the plant material.

The ‘force’ functionality in DARwin was used for the initial selection step to include 11 accessions with interesting phenotypic variability such as early budbreak date, red kernel, or good adaptation to French growing conditions such as the cultivars belonging to Protected Designations of Origin. These accessions, containing a relatively low level of genetic diversity, are part of the ‘Western Europe and America’ cluster (except ‘Lara’ which is admixed).

The second step selected 39 accessions with maximum of genetic diversity, including four from the ‘Western Europe and America’ cluster and 35 from the ‘Eastern Europe and Asia’ cluster. Notable were ‘UK-series’ accessions from a probable center of origin, and the three ‘PI-series’ from Poland, Afghanistan and Russia [60]. Also included were accessions from Iran, China, Greece, India, Romania and Bulgaria. Additional selections for the core collection were ‘Sexton’, with Chinese parentage, and a tree with large leaves that could be interesting for ornamental use. Seventy percent of the core collection is comprised of accessions from the ‘Eastern Europe and Asia’ cluster, reflecting the greater diversity available in this cluster.

## Conclusions

This study used SSR markers to assess the genetic diversity and structure of the INRA walnut germplasm collection. Resulting knowledge and the core collection constructed will be useful for rational and economically sustainable management of INRA collection. In addition, this information will provide a valuable and fruitful tool for walnut breeders in selecting new cultivars and identifying promising parents for crossbreeding. A highly promising hybrid from the second INRA improvement program, which is currently under evaluation, illustrates this point well. This hybrid is a cross between ‘Fernor’ and ‘Shinrei’ from ‘Western Europe and America’ and ‘Eastern Europe and Asia’ clusters respectively and demonstrates the efficiency of choosing parents based on their cluster membership. Use of the knowledge developed in this study should increase the efficiency of the new French breeding program in addressing challenges to the walnut industry such as adaptation to climate change and resistance to new pests and diseases, while also increasing yield and kernel quality. This work also highlights the importance of collecting germplasm in, or close to, countries of the crop’s center of origin.

In addition, assessment of the genetic diversity of INRA walnut collection could be used to define additional core collections suitable for further analysis, such as a genome-wide association study. These collections would be characterized by unique and great phenotypic variability specifically regarding particular traits of interest such as early budbreak and bloom dates, nut and kernel quality, and tolerance to pests and diseases.

## Supporting information

S1 Fig Detection of the number of populations K using plateau criterion (Pritchard et al., 2000) and ΔK method (Evanno et al., 2005). 217 J. regia accessions, K = 2
(TIFF)

S2 Fig Graphical method allowing the detection of the number of populations K using ΔK (Evanno et al., 2005). 63 ‘East. Eur. and Asia’ accessions, K = 3.
(PDF)

S3 Fig Graphical method allowing the detection of the number of populations K using ΔK (Evanno et al., 2005). 127 ‘West. Eur. and Am.’ accessions, K = 3.
(PDF)

S1 Table List of accessions studied. (XLSX)

S2 Table SSR allele-specificity among *Juglans* species. (XLSX)

S3 Table Table summarizing the results using the Evanno method (output of Structure Harvester), based on the analyses of the 217 *J. regia* accessions. (XLSX)

S4 Table List of *J. regia* accessions with their memberships to clusters (K = 2), subclusters (K = 3) and core collection. (XLSX)

S5 Table Table summarizing the results using the Evanno method (output of Structure Harvester), based on the analyses of the 63 Eastern Europe and Asia accessions. (XLSX)

S6 Table Table summarizing the results using the Evanno method (output of Structure Harvester), based on the analyses of the 127 Western Europe and America accessions. (XLSX)

S7 Table Genetic diversity estimations of the clusters found with STRUCTURE K = 2. (XLSX)

S8 Table Pairwise FST among populations identified with STRUCTURE K = 2. (XLSX)

S9 Table Private alleles among clusters for *J. regia* (K = 2). (XLSX)

S10 Table Raw data set necessary to replicate the study. (XLSX)
