# Supplementary figures and images for: Analysis of genetic diversity and structure in a worldwide walnut (Juglans regia L.) germplasm using SSR markers
Source: PLoS One. 2018 Nov 27;13(11):e0208021. doi: 10.1371/journal.pone.0208021 (PMC6258541; doi:10.1371/journal.pone.0208021)

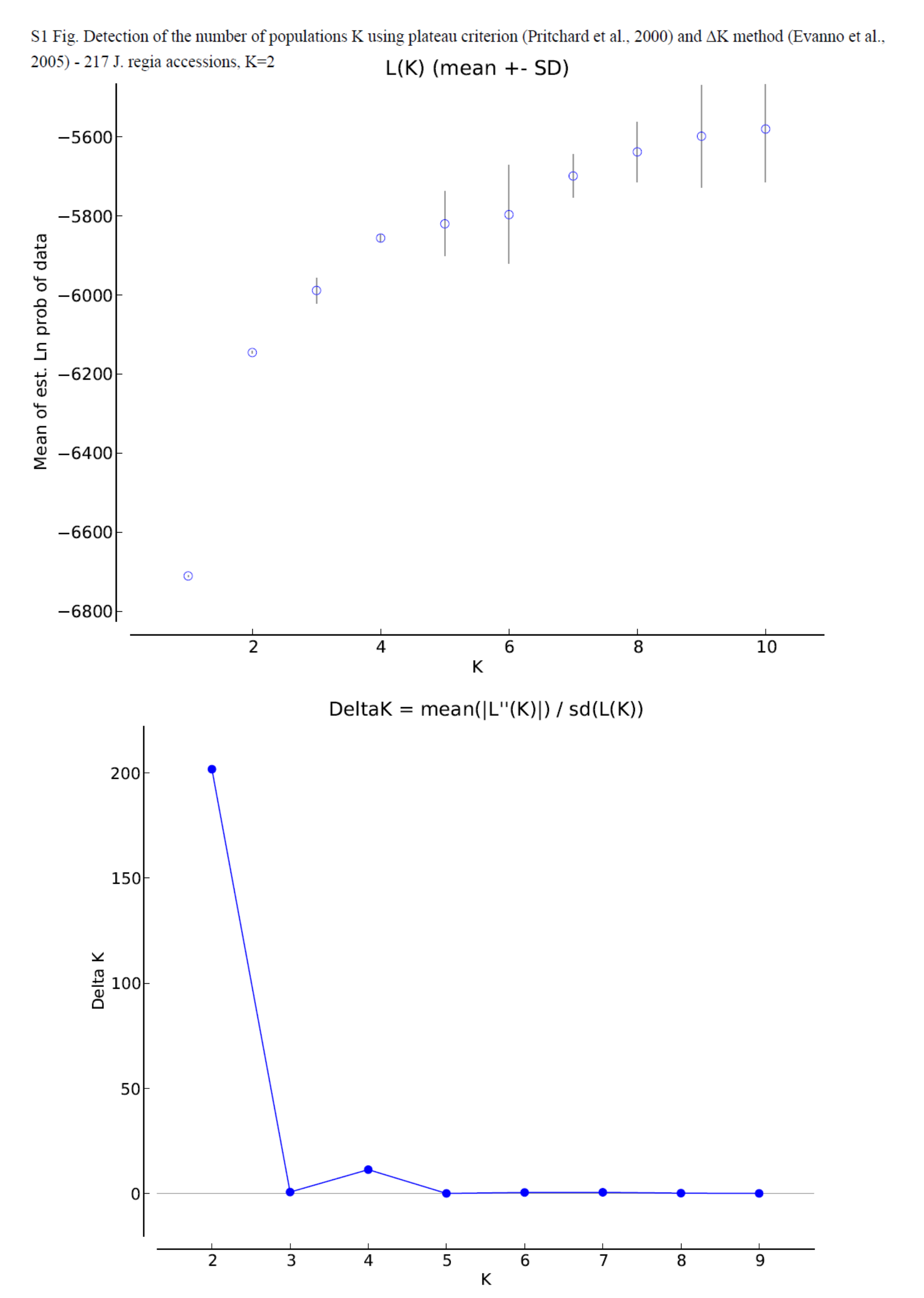

Supplement: S1 Fig — 217 J. regia accessions, K = 2 (TIFF) [file pone.0208021.s001.tiff]
