# Supplementary material for: Analysis of genetic diversity and structure in a worldwide walnut (Juglans regia L.) germplasm using SSR markers
Source: PLoS One. 2018 Nov 27;13(11):e0208021. doi: 10.1371/journal.pone.0208021 (PMC6258541; doi:10.1371/journal.pone.0208021)

S3 Fig. Graphical method allowing the detection of the number of populations K using ΔK (Evanno et al., 2005)

127 'West. Eur. and Am.' accessions, K=3

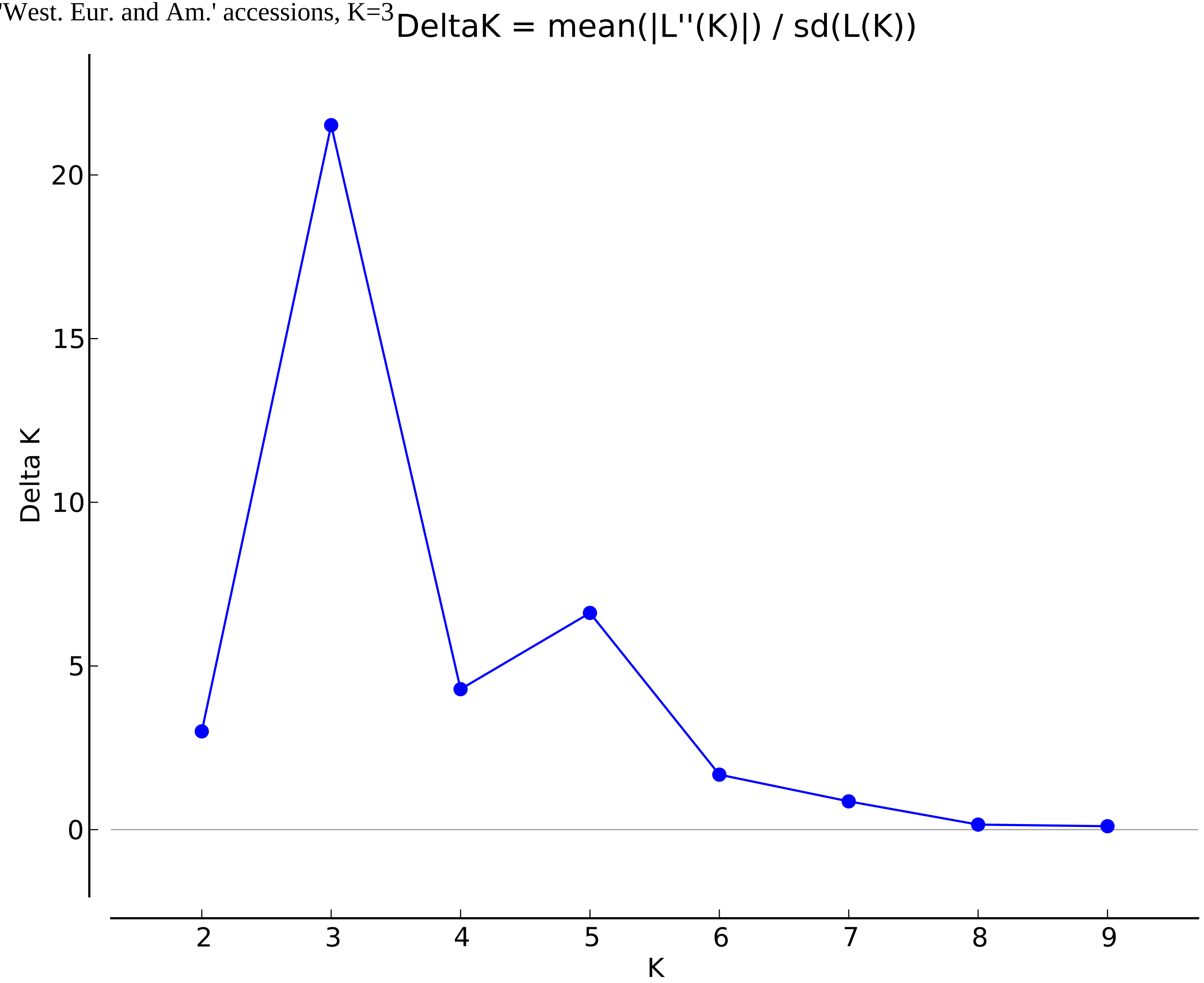

Supplement: S3 Fig — 127 ‘West. Eur. and Am.’ accessions, K = 3. (PDF) [file pone.0208021.s003.pdf]
